# The RNA helicase A in malignant transformation

**DOI:** 10.18632/oncotarget.7377

**Published:** 2016-02-14

**Authors:** Marco Fidaleo, Elisa De Paola, Maria Paola Paronetto

**Affiliations:** ^1^ Department of Movement, Human and Health Sciences, University of Rome “Foro Italico”, Rome, Italy; ^2^ Laboratory of Cellular and Molecular Neurobiology, CERC, Fondazione Santa Lucia, Rome, Italy

**Keywords:** RHA helicase, genomic stability, cancer

## Abstract

The RNA helicase A (RHA) is involved in several steps of RNA metabolism, such as RNA processing, cellular transit of viral molecules, ribosome assembly, regulation of transcription and translation of specific mRNAs. RHA is a multifunctional protein whose roles depend on the specific interaction with different molecular partners, which have been extensively characterized in physiological situations. More recently, the functional implication of RHA in human cancer has emerged. Interestingly, RHA was shown to cooperate with both tumor suppressors and oncoproteins in different tumours, indicating that its specific role in cancer is strongly influenced by the cellular context. For instance, silencing of RHA and/or disruption of its interaction with the oncoprotein EWS-FLI1 rendered Ewing sarcoma cells more sensitive to genotoxic stresses and affected tumor growth and maintenance, suggesting possible therapeutic implications.

Herein, we review the recent advances in the cellular functions of RHA and discuss its implication in oncogenesis, providing a perspective for future studies and potential translational opportunities in human cancer.

## INTRODUCTION

RNA helicase A (RHA) is a DNA/RNA helicase involved in all the essential steps of RNA metabolism, such as transcription, pre-mRNA splicing, translation and ribosome biogenesis [1, 2, 3]. RHA was first purified in 1991 from calf thymus nuclei for its DNA helicase activity [4], and subsequently described as the most abundant and stable RNA helicase present in HeLa nuclear extracts [5, 6]. Also known as DEAH (Asp-Glu-Ala-His) box helicase 9 (DHX9), or nuclear DNA helicase II (NDHII), RHA belongs to the DHX helicase family, characterized by a DEAH amino acid sequence in the motif II of the helicase domain (signature motif) and differing from the helicase domain of the DDX helicase family, which contains a DEAD (Asp-Glu-Ala-Asp) amino acid sequence [7].

RHA orthologous proteins have been identified in *Drosophila* (maleless, MLE) [8], in *C. elegans* (RHA-1) [9] and in mouse (RHA) [10]. The fly MLE displays 50% of amino acid identity and 85% similarity with human RHA and is involved in dosage compensation for male development [8]. In particular, MLE increases two fold the transcription of the single X chromosome in male gnats thus equalizing the mRNA levels with those of females, which contain two X chromosomes [8]. The *C. elegans* RHA-1 displays about 60% of similarity with both human RHA and *Drosophila* MLE and is involved in gene silencing. Mouse and human RHA proteins display high levels of homology, with 93% of amino acid identity [11].

Genetic ablation models performed in different species clearly highlighted the essential role played by RHA helicase. Mutations in the fly *mle* lead to selective death of male flies that cannot pupate and die as larvae [12, 6]. *Rha-1* mutations in worms produce transcriptional de-silencing at restrictive temperature causing defects in germ cell proliferation [9]. Homozygous *rha* mutation in mice determines apoptosis of embryonic ectodermal cells during gastrulation and early embryonic lethality in both sexes [10]. Mice carrying *rha* mutations on one allele are viable, albeit they express lower protein level than wild type [13]. In humans, mutations in *RHA* and alteration in RHA expression are found in a wide range of cancers, suggesting that non-functional RHA protein is involved in malignant transformation [14, 15]. For instance, the gene encoding RHA was identified as one of ten genes displaying recurrent mutations that were highly correlated with pathway deregulation and patient survival in lung adenocarcinoma [15]. Nevertheless, several aggressive tumors overexpress RHA [16]. Importantly, RHA participates in the maintenance of genomic stability [17, 18]. Moreover, in Ewing sarcoma cells RHA confers resistance to UV light irradiation and chemotherapeutic treatment, while genotoxic drug treatments able to reduce RHA expression can inhibit tumor growth [19]. These observations on a positive role played by RHA in Ewing sarcoma are in line with the finding that RHA down-regulation sensitizes lymphomas to chemotherapeutic treatment [20]. Taken together, these studies suggest that the role of RHA in cancer transformation and in chemotherapy resistance may strongly depend on the cellular context in which transformation occurs.

Despite the growing interest in RHA helicase for therapeutic purpose, its physiological role has not been completely elucidated yet. In this review, we discuss the functional properties of RHA in signaling and RNA metabolism. In particular, we highlight recent advances and new insights on RHA-protein and RHA-RNA molecular interactions to draw an updated picture of its involvement in malignant transformation and in the maintenance of genomic stability.

## RHA PROTEIN STRUCTURE AND DOMAINS

**Figure 1:**
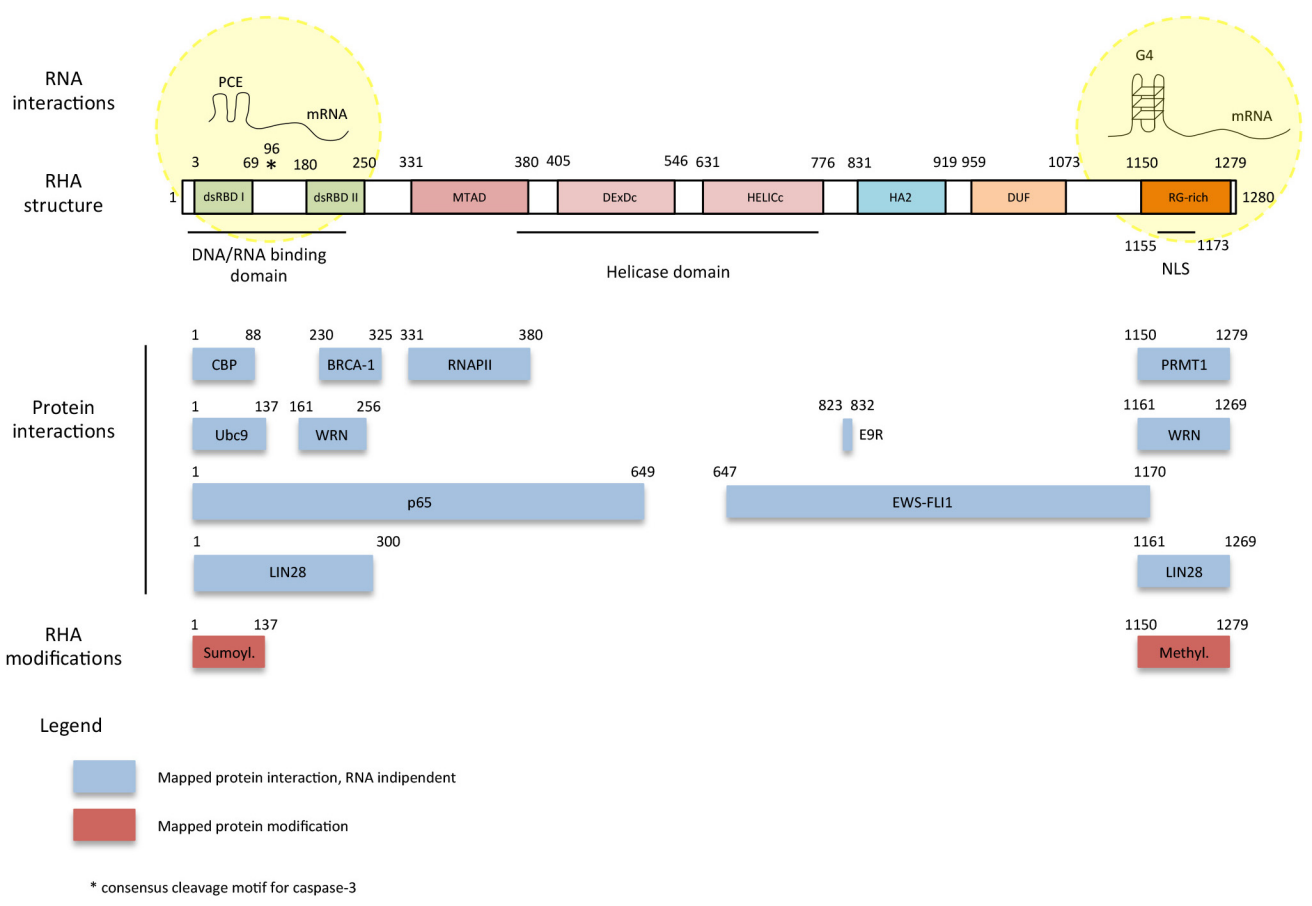
Scheme of RHA protein domains and physical interactions RHA is a 140 KDa protein formed by eight domains. The N-terminal part of the protein is characterized by two repeats of double-stranded RNA-binding domain (dsRBD I and dsRBD II) and the minimal transactivation domain (MTAD), while the central part contains a conserved ATPase-dependent helicase domain, a Helicase-Associated domain 2 (HA2) and a Domain of Unknown Function (DUF). The C-terminus is formed by repeated arginine and glycine (RG) residues (RG-rich domain). NLS indicates the lysine-rich nuclear localization signal. RHA is phosphorylated in a RNA-depended manner by the DNA-dependent protein kinase (DNA-PK). Phosphorylation determines RHA subnuclear localization (nucleoli exclusion) and correct activity. Moreover, RHA arginine-rich C-terminal region interacts with arginine methyltransferase 1 (PRMT1). The N-terminus of RHA (including dsRBD) can undergo sumoylation. E9R is the peptide corresponding to amino acids 823-832 of RHA which binds EWS-FLI1 and inhibits RHA/EWS-FLI1 interaction.

The gene encoding human RHA maps to the major susceptibility locus for prostate cancer at chromosome band 1q25, while its pseudogene is located on chromosome 13q22 [21]. The *RHA* gene encodes a 140 KDa protein formed by eight domains (Figure 1). The N-terminal part of the protein is characterized by two repeats of double-stranded RNA-binding domain (dsRBD I and dsRBD II) and by the minimal transactivation domain (MTAD) [1]. RHA dsRBDs display specificity for dsRNA and a limited affinity for single-stranded DNA [1]. Moreover, dsRBDs domains are able to bind the Post-transcriptional Control Elements (PCEs) in the 5′untranslated regions (UTR) of specific mRNAs thus modulating their translation [3]. The central part of the protein contains a conserved ATPase-dependent helicase domain, formed by a DEAD-like helicase superfamily ATP binding domain (DExDc) and a Helicase superfamily C-terminus domain associated with DExH/D box proteins (HELICc), a Helicase-Associated domain 2 (HA2), and a Domain of Unknown Function (DUF) [1, 22] (Figure 1). The helicase domain is required for ATP binding, hydrolysis, nucleic acid binding and unwinding [23, 24]. The C-terminus of RHA is formed by repeated arginine and glycine (RG) residues (RG-rich domain) [1]. In general, RGG-boxes cooperate with other domains to achieve and increase affinity for nucleic acids and are involved in RNA-based binding to G quadruplex structures [25, 26, 1, 27]. A lysine-rich nuclear localization signal (NLS) is present in the mammalian RHA between the helicase domains [28], which is not present in the *Drosophila* maleless protein [8] and in the RHA from *Caenorhabditis elegans* [29]. Moreover, a highly conserved NLS of 19 amino acid residues has been identified in the C-terminus of RHA [30] revealing that its nuclear import is Ran-dependent and mediated by importin-alpha/beta.

RHA preferentially binds to single-strand (ss) DNA, which occurs *in vivo* at replication forks, transcription bubbles, promoters, or nuclear matrix attachment sites on chromatin loops. RHA unwinds DNA and RNA in 3′ to 5′ direction [5, 31]. Biochemical studies confirmed *in vitro* the specificity of the dsRBDs for dsRNA, and the preferential binding of the RGG-boxes to single strand nucleic acids [1]. Importantly, proteolytic deletion studies documented that neither disruption of the RGG-box nor of the two dsRBDs abolished the unwinding activity, although their proteolytic removal diminished the nucleic acid-stimulated ATPase activity of RHA [1]. Thus, single-stranded/double-stranded nucleic acid contacts with the RGG domain and the dsRBDs might trigger the activation of the ATPase/helicase activity, and cooperation between the RGG domain and the dsRBDs might represent the first step in the enzymatic activity of RHA. The kinetic and molecular mechanism involved in RHA RNA unwinding/rewinding activity were characterized by single-molecule Förster Resonance Energy Transfer (smFRET), a technique that enables detection of unwinding by a single RHA on a duplex RNA molecule [32]. The smFRET of wild type and dsRBD-deleted RHA proteins showed that the dsRBDs increase the binding affinity and contribute to the stability of RHA binding to dsRNA [32].

Finally, RHA, by directly interacting with specific mRNA structures (i.e. post-transcriptional control element, PCE, and G-quadruplex, G4) can modulate mRNA translation of gene involved in oncogenesis, such as the proto-oncogene *JUND*, encoding a member of the AP-1 transcription factor family [3].

Several post-translational modifications modulate RHA functions. RHA is phosphorylated in a RNA-dependent manner by the DNA-dependent protein kinase (DNA-PK) and phosphorylation determines its subnuclear localization, with exclusion from the nucleoli, and affects its activity [28]. Moreover, RHA methylation by the arginine methyltransferase 1 (PRMT1) in the RGG domain determines its nuclear import [33]. Lastly, the N-terminus of RHA (including dsRBD) undergoes sumoylation, which affects RHA-mediated transcriptional activity [34]. Nevertheless, whether or not these post-translational modifications also affect RHA nucleic acid binding activity has not been investigated yet.

## RHA AND TRANSCRIPTIONAL REGULATION

RHA was initially proposed as an intermediate factor bridging the RNA polymerase II (RNAPII) to the cAMP response element-binding (CREB) binding protein (CBP)/p300 [35]. In particular, the interaction of RHA with RNAPII is mediated by aromatic residues (e.g. tryptophan) contained in the minimal transactivation domain (MTAD, residues 331-380) [36], while the interaction with CBP/p300 is mediated by the residues 1-88 [37] (Figure 1). Thus, RHA, acting as a hinge, would transmit regulatory signals to RNAPII by bridging other interacting proteins. In this way, the interaction with multiple regulatory proteins becomes functional to display different roles in multiple aspects of gene regulation, thus having an impact on cell proliferation, differentiation and even transformation [35].

The interaction of RHA with CBP is strategically relevant in oncogenesis. In fact, CBP links DNA-binding factors to the transcriptional machinery and is implicated in the regulation of the expression of genes involved in malignant transformation, such as *c-MYC*, *JUN*, *FOS*, transforming viral proteins (such as E1A, E6 and large T antigen) and tumor-suppressor proteins (such as p53, E2F, Rb, Smads, RUNX and BRCA1) [38]. Remarkably, mutations in the CBP-binding region of RHA occur in several human tumors [39] and were reported to strongly affect regulation of gene expression.

CBP/p300 up-regulates the level of the breast cancer specific gene *BRCA-1* [40]. *BRCA1* is a human tumor suppressor gene that plays critical roles in maintenance of genomic stability [41]. *BRCA1* is expressed in breast and other tissues, where it helps to repair damaged DNA [42] by forming a large multiprotein complex known as the BRCA1-associated genome surveillance complex with DNA damage sensors and other tumor suppressors [40]. BRCA1 inherited mutations in *BRCA1* or *BRCA2* predispose to breast, ovarian, and other cancers [43, 44]. It is possible that mutations in the CBP-binding region of RHA affect CBP/p300 co-factor transcriptional activity, thus impairing the transcription of *BRCA-1*. Similarly to CREB-dependent transcriptional activation, RHA also activates transcription by interacting directly to BRCA1 [45]; BRCA1-RHA interaction involves the residues 230-325 and allows, as in the case of CBP, the concomitant association with RNAPII [37] (Figure 1). Breast cancer-related BRCA1 mutants display low ability to bind RHA thus reducing BRCA1 tumor suppressor activity and promoting cancer growth [45].

Recently, the BRCA1/RHA interaction has emerged to play a fundamental role also in microRNA (miRNA) maturation. Cancer transformation is strongly related to impaired miRNA regulation and/or dysregulation of miRNA processing [46], while abnormalities in miRNAs contribute to the pathogenesis of human tumors [47]. Importantly, the expression of several miRNAs is dysregulated in BRCA1/2 mutated cells [48]. BRCA1 interacts with the DROSHA microprocessor complex and regulates the processing of a small set of precursor and mature miRNAs, including let-7a-1, miR-16-1, miR-145, and miR-34a [49]. Accordingly, RNA immunoprecipitation (RIP) experiments showed that both BRCA1 and RHA associate with pri-let-7a-1, miR-16-1, miR-145, and miR-34a, while RHA knockdown suppressed the processing of the pri-miRNAs let-7a-1, miR-16-1, miR-145, and miR-34a [49]. These findings open the possibility that RHA participates in miRNA processing in complex with BRCA1 and that aberrant regulation of miRNA processing by mutations in BRCA1 and/or RHA contribute to oncogenic transformation.

The proto-oncoprotein p65 was initially found associated with RHA by a yeast two-hybrid screening [50] and the direct interaction was confirmed later by *in vitro* and *in vivo* experiments [50]. RHA-p65 interaction involves the N-terminal region of RHA (1-649 aa; Figure 1) and the Groucho-interacting region (GIR) of p65, located between the transactivation (TA) domain 1 and the TA1-like motifs of p65 protein [50]. Hence, as for CBP and BRCA1, the interaction with RHA could contribute to RNAPII recruitment for the formation of a transactivation complex. RHA binding activates NF-κB-mediated transcription, while RHA knockdown reduce the NF-κB-mediated gene expression [50]. The ATP-binding and helicase activity of RHA are required for the transcriptional activation mediated by NF-κB. Interestingly, the TA1-like and TA1 domains of p65 can also bind CBP/p300. Thus interaction of RHA with p65/CBP/p300 may form a large multimolecular complex driving gene expression. This NF-κB-dependent gene expression program is inhibited by both RHA knockdown or dominant negative mutants of RHA (lacking the ATP-binding and helicase activity) while it is increased by RHA overexpression [50]. Since NF-κB signaling is involved in tumorigenesis [51], and several tumors show upregulation of RHA expression [16], the increase of RHA may affect NF-κB-mediated transcription thus contributing to cancer transformation and drug resistance.

RHA undergoes sumoylation both *in vitro* and *in vivo*: Ubc9, the E2-like enzyme specific for small ubiquitin-like modifier 1 (Sumo-1), interacts and sumoylates the N-terminal domain of RHA (residues 1-137) [34]. The functional significance of RHA sumoylation remains unknown. Since Ubc9 and the SUMO pathway revealed a major role in nuclear architecture and in chromosome condensation and segregation [52], these processes might require RHA activity. In support of this hypothesis, in G2/M phase, RHA stably associates with the toposome, a multisubunit complex formed by the topoisomerase IIα with two ATPase/helicase proteins (RNA helicase A and RHII/Gu), one serine/threonine protein kinase (SRPK1), one HMG protein (SSRP1), and two pre-mRNA splicing factors (PRP8 and hnRNP C) [53]. The interaction between RHA and topoisomerase IIα requires Ubc9 [54]. Topoisomerase IIα is a multifunctional enzyme that catalyzes the relaxation of supercoiled DNA, decatenation of interlinked DNA and unknotting of intramolecularly linked DNA by passing a DNA helix through a transient double-strand break in a second helix [55]. Ubc9/RHA interaction could serve as mediator of RHA/topoisomerase IIα interaction (independently of its Sumo-1 conjugation activity), thus highlighting the involvement of RHA in topology of chromatin DNA, influencing both chromosome condensation and transcriptional activity [54]. Indeed, the interaction of topoisomerase IIα with RHA occurs in an RNase-sensitive manner [54]. Very recently it has been observed that up-regulation of Ubc9 promotes migration and invasion of lung cancer [56]; the fact that high levels of RHA have been described in lung cancer and RHA upregulation correlated with high grade tumors [16], strengthen the hypothesis of a neoplastic role of Ubc9/RHA interaction.

## RHA AND POST-TRANSCRIPTIONAL REGULATION OF GENE EXPRESSION

A role for RHA in post-transcriptional regulation of HIV type 1 has been described [57]. In particular, the dsRBD domains of RHA are involved in the recognition of PCE, a long and highly structured 5′UTR belonging to a class of mRNA formed by naturally unspliced templates [3]. PCE is formed by 150-nucleotide displaying two functionally redundant stem-loop structures (called “A” and “C”). PCEs have been identified in several virus such as avian spleen necrosis virus (SNV), Mason-Pfizer monkey virus and HIV and some naturally intronless cellular genes [3]. Bioinformatics analyses predicted about 200 human genes containing PCEs, including the proto-oncogene *JUND* [3]. Importantly, mutations in the PCE stem-loop inhibit translation and do not allow RHA binding [3]. On the other side, mutations in the conserved lysine residues of dsRBD I or II of RHA reduce, but do not abolish, RHA affinity for PCE structures. Mutations affecting both the dsRBD domains of RHA completely abolish RHA translational activity of retroviral PCE-containing RNAs [22]. Interestingly, RHA and PCE RNAs coprecipitate from nucleoplasm thus suggesting an early interaction with target mRNAs immediately after transcription [3]. The translation of PCE-containing mRNAs begins with a cap-dependent mechanism [58]. PCE structures are located at the distal 5′UTR and represent a barrier for efficient ribosome scanning; the interaction between RHA and PCE induces RNA-protein and RNA-RNA rearrangements that allow polyribosomes access and increases the rate of protein synthesis [3]. Indeed, nascent RNA can form RNA-based G4 structures that might have a role in translational repression of proto-oncogenes. For example, human *NRAS* proto-oncogene has thermodynamically stable RNA G4 structure in the 5′ UTR which exhibited a role in gene modulation in a cell-free translation system [59]. The DHX36 helicase, a member of DEAH-box family, has been described to solve RNA-G4 structure, but whether or not RHA displays the same activity has not been unraveled yet [60]. Thus, the activity of RHA in unwinding G4 structures seems to be involved in malignant transformation.

Consistent with a role in translational regulation, RHA was described to interact directly with Lin28, an evolutionary conserved RNA binding protein that acts as a repressor of miRNA biogenesis and as a positive regulator of translation of selected transcripts [61, 62]. In human embryonic stem cells Lin28 facilitates the expression of the pluripotency factor Oct4 at the post-transcriptional level: binding of Lin28 to Oct4 mRNA was enhanced by Lin28-RHA interaction while RHA depletion impairs Lin28-dependent stimulation of Oct4 translation [61, 62]. Thus, it is possible that Lin28 target mRNAs may display a common structural or sequence features that reduce their translational efficiency. The binding of Lin28 and subsequent recruitment of RHA to these mRNAs would overcome the inhibition by removing the inhibitory structures, thus allowing efficient translation. Importantly, a mutant Lin28 that still binds RNA but is unable to interact with RHA, acts as a dominant-negative inhibitor of Lin28-dependent stimulation of translation [63]. Similarly, knockdown of RHA in human lung fibroblasts prevents formation of polysomes on collagen mRNAs and dramatically reduces synthesis of collagen protein, without affecting the level of the mRNAs [64].

Given the reported findings, we can conclude that by unwinding RNA secondary structures RHA may help ribosome assembly on target mRNAs to efficiently achieve their translational elongation.

## RHA AND GENOMIC INSTABILITY

The integrity of the genome is challenged by a variety of exogenous and endogenous agents. Accurate DNA replication and DNA repair are crucial for the maintenance of genome stability. Failure of these processes is a major source of DNA damage in cells and a leading cause of the accumulation of mutations. Thus, genomic instability is one of the main mechanisms underlying malignant transformation [65]. A large body of evidence suggests that conflicts between the transcription and replication machineries are a major source of the observed defects. In particular, formation of co-transcriptional RNA:DNA hybrid structures, known as R-loops, may significantly contribute to the genomic instability [66]. Moreover, non-canonical (i.e. non-B) DNA structures that can form transiently during replication and transcription have the potential to trigger the formation of intra-molecular triplex DNA (H-DNA), which have been shown to block replication *in vitro* and to promote DNA double-strand breaks (DSBs) [67, 68]. Thus these non-B DNA-structures (like repetitive DNA motifs, short tandem repeats, inverted repeats, alternating purine-pyrimidine tracts, and G-rich sequences) induce genetic instabilities in the form of deletions, translocations and single-base substitutions [69].

**Figure 2:**
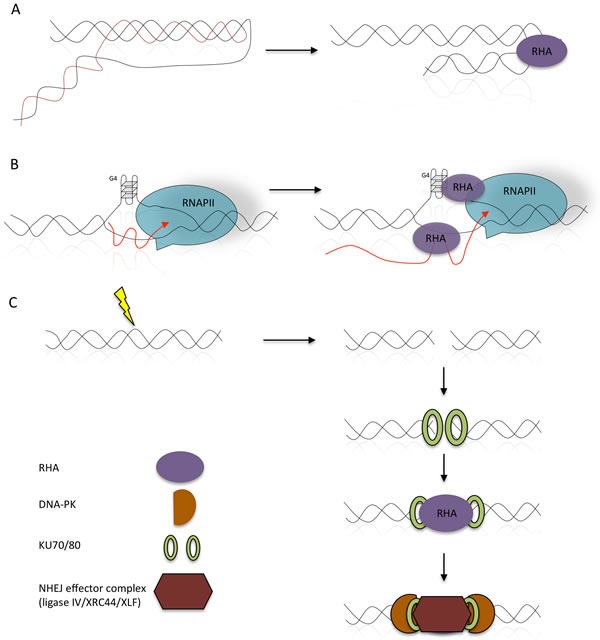
Involvement of RHA in the processing of R-loops and H-DNA structures **A.** The helicase activity of RHA resolves the mutagenic H-DNA structures, thus preserving genetic instability (arranged from [17, 97]). **B.** Formation of co-transcriptional RNA:DNA hybrid structures, known as R-loops, may significantly contribute to the genomic instability. Stalled transcription bubbles create negative supercoils that may allow R-loop formation by invasion of the nascent transcript. R-loop formation can get even more prominent when the displaced DNA strand is G-rich and forms a G-loop. RHA removes both R-loops and DNA-based G4-structures thus contributing to transcription and preventing genomic instability. **C.** RHA participates in non-homologous end-joining of H-DNA-induced DSBs by protecting the free ends, and recruiting repair proteins, thus limiting genomic instability (arranged from [17, 97]).

Many observations suggest the involvement of RHA in several mechanisms aimed at preserving genomic stability [17, 60]. Si-RNA mediated RHA depletion increased the frequencies of mutations induced by H-DNA [17]. On the other hand, loss of RHA results in early senescence in fibroblasts [18]. Moreover, intra-molecular triplex DNA (H-DNA) structures are bound and resolved by RHA protein in human osteosarcoma U2OS (Figure 2A) [17]. Remarkably RHA helicase activity contributes to RNA:DNA hybrid unwinding, thus allowing R-loops to be resolved (Figure 2B) [60]. Interestingly, formation of R-loops is facilitated by G-rich sequences and transcriptional supercoiling, and RHA preferentially unwinds R-loops and DNA-based G-quadruplexes [60]. Thus, by unwinding these structures RHA may significantly contribute to transcriptional activation and to the maintenance of genomic stability.

Upon genotoxic insults, γ-H2AX accumulates in transcriptionally active chromatin foci and binds RHA in a RNA dependent manner [70]. γ-H2AX-RHA interaction sequestrates RHA in stalling transcription bubble and slows down RNA synthesis [70]. Interestingly, genotoxic stress greatly impacts the phosphorylation state of RNAPII and its degradation [71]. In particular two covalent modifications are associated with this phenomenon. Genotoxic stress induces first the hyper-phosphorylation of RNAPII carboxy terminal domain (CTD), followed by RNAPII ubiquitylation [72, 73]. Ubiquitylation in turn accelerates proteasome-dependent degradation of the polymerase [72]. Thus, the dynamics of protein assembly/disassembly at sites of DNA breaks and their post-translational modifications allows repair factors recruitment and orchestrates the interplay between the DNA damage response and RNA synthesis. To this purpose, inhibition of transcription upon DNA damage could offer a time window for the recruitment of the repair machinery to the sites of break.

Ku antigen is a DNA-binding checkpoint kinase involved in DNA damage signaling and dsDNA unwinding activity. It consists of two subunits of 80 and 70 kDa involved in DNA double-strand break repair and V(D)J recombination [74]. Immunoprecipitation experiments documented that Ku interacts with RHA in a RNA-dependent manner in HeLa cells [28]. The DNA unwinding activity of RHA might facilitate the entry of Ku into chromosomal binding sites nearby the breaks (Figure 2C). Interestingly both Ku protein and RHA have been described as autoantigens in patients suffering from systemic lupus erythematosus (SLE) [75, 76], suggesting the hypothesis of cooperative activity of the two proteins. Moreover, some autosera against Ku antigen cross-react with other RNA-binding proteins and components of snRNPs [77], thus all these proteins may form a functional complex *in vivo*. Another intriguing possibility is that RHA, as well as Ku proteins, might recruit noncoding RNAs on the DNA double-strand breaks, and that the crosstalk between noncoding RNAs and the DDR might provide a more efficient and accurate DNA repair facilitating the maintenance of genomic stability [78].

RHA also interacts with the Werner Syndrome Helicase (WRN) [79], which in turn interacts with Ku [80]. WRN contains both a 3′ → 5′ helicase and a 3′ → 5′ exonuclease activity. The exonuclease domain of WRN binds RHA *via* the dsRBD II and the RGG-box, thus occluding the two domains from DNA binding, and leads to inhibition of the DNA helicase activity of RHA [79]. On the other hand, dsRBD II and the RGG-box of RHA directly stimulate the 3′-5′ exonuclease activity of WRN, possibly in a manner similar to how they stimulate the helicase activity of RHA [79]. Importantly, defective WRN DNA helicase causes the Werner syndrome, a rare autosomal recessive genetic disorder manifested by the symptoms of premature aging, such as atherosclerosis, osteoporosis, diabetes mellitus type II, cataracts, and genomic instability with an increased incidence of tumor formation [81]. During interphase, a fraction of WRN and RHA co-localizes at centrosomes together with γH2AX [82], and both enzymes interact with the mediator of homologous recombination BRCA1 [45]. Importantly, centrosome amplification occurs frequently in almost all types of cancer, and is considered as the major contributing factor for chromosome instability in cancer cells. Collectively these findings suggest that RHA plays a role in promoting the processing function of WRN necessary for maintaining genomic stability. Since RHA physically interacts also with the SMN (survival of motor neurons) protein [83], that in turn is associated with small nuclear and nucleolar ribonucleoproteins, spliceosomal proteins and with the RNA polymerase II, we may reasonably hypothesize that *via* these physical links, RHA can be recruited to the DNA and to the RNA processing machineries to play a role in both supervising the genomic integrity and processing RNAs.

## RHA AND CANCER

Deregulation of the mechanisms guiding programmed cell death plays an important role in the pathogenesis and progression of cancer, as well as in tumor response to therapeutic intervention. Evading apoptosis through genetic mutation is considered a hallmark of cancer malignancy [84]. Notably, defective apoptosis not only allows tumorigenesis but can also lead to resistance to chemotherapy. As mentioned above, mutations in the gene encoding RHA and alterations in RHA expression are found in a wide range of cancers [16, 14, 15]. This observation raises the hypothesis that non-functional RHA protein is involved in malignant transformation. Supporting this notion, an RNAi screen recently identified the gene encoding RHA as a regulator of the sensitivity of lymphoma cells to the chemotherapeutic agent ABT-737 [20]. Resistance of lymphoma cells to ABT-737 is mainly driven by the upregulation of the MCL-1 pro-survival protein. Loss of RHA improved ABT-737 sensitivity by intensifying MYC oncogene-induced replicative stress and triggering induction of the p53 apoptotic program [20].

In a different setting, however, RHA was shown to contribute to tumor suppression. In neuronal cells, RHA interacts with the tumor suppressor KIF1Bβ and it is necessary for KIF1Bβ-mediated apoptosis in NGF-limiting conditions [39]. Abnormal NGF signaling has been linked to nervous system tumors such as neuroblastoma, medulloblastoma, and pheochromocytoma [85]. Low expression of KIF1Bβ correlated with poor prognosis and reduced survival of patients with neuroblastoma, providing evidence that KIF1Bβ is a neuroblastoma tumor suppressor. Importantly, in KIF1Bβ-deficient neuroblastoma tumors, RHA nuclear localization is impaired, leading to an accumulation of the helicase in the cytoplasm. This suggests that loss of KIF1Bβ may impair NGF-deprived apoptosis due to mislocalization of RHA [39] and predispose to neuroblastoma formation. Thus, the opposite roles played by RHA in human cancers strongly suggest that its activity is redirected towards different functions depending on the molecular partners with which it interacts and the cellular context in which tumorigenesis occurs.

## RHA AND EWING SARCOMA

**Figure 3:**
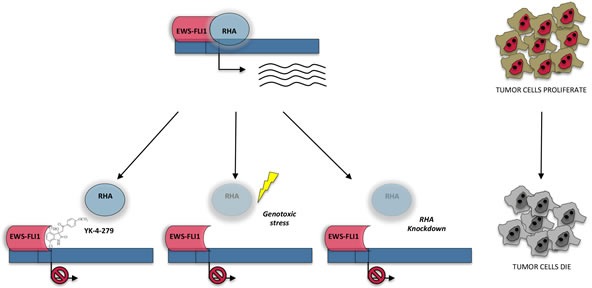
Interplay between RHA and EWS-FLI1 in the regulation of gene expression of Ewing sarcoma In Ewing sarcoma cells RHA, acting as hinge between EWS-FLI1 and RNAPII, is involved in EWS-FLI1 mediated gene expression. Inhibition of RHA-EWS-FLI1 by the small molecule YK-4-279 inhibits EWS-FLI1 transcriptional activity. Similarly, downregulation of RHA, induced either in response to genotoxic stress which triggers the selection of an alternative splicing isoform targeted to NMD (like) or by RNA interfering, negatively affects EWS-FLI1 target gene expression. Thus, targeting RHA or RHA-EWS-FLI1 interaction represents a valuable tool to affect ES cell viability and proliferation.

RHA was identified as a molecular partner of the oncoprotein EWS-FLI1, which is essential for growth and maintenance of a subset of Ewing sarcoma [86]. EWS-FLI1 is an oncoprotein found in about 85% of Ewing sarcoma. It results from the fusion between the Ewing sarcoma breakpoint region 1 gene (*EWSR1*) on chromosome 22 and the Friend leukemia virus integration site 1 gene (*FLI1*) on chromosome 11 [87]. EWS-FLI1 is characterized by the N-terminus of EWS, which is a potent transcriptional activation domain, fused to the DNA-binding domain of the transcription factor FLI1. This chimeric protein guides an aberrant transcription program that promotes oncogenesis [88, 89]. RHA (residues 647-1170) interacts with EWS-FLI1 (Figure 1) and stimulates the transcriptional activity of EWS-FLI1-regulated promoters in ES cells [86] (Figure 3). Strikingly, RHA expression in mouse embryonic fibroblasts stably transfected with EWS-FLI1 enhances anchorage-independent growth more than EWS-FLI1 alone [86]. This observation suggests that RHA works as a transcriptional cofactor to enhance EWS-FLI1 function, like for CBP and BRCA1 [45, 35, 37]. In addition, unlike its interaction with CBP and BRCA1, the interaction of EWS-FLI1 with RHA also affects modulation of pre-mRNA processing, contributing to splicing isoforms involved in oncogenesis [90]. Thus, RHA binding to EWS-FLI1 is important for its oncogenic function and for the accomplishment of oncogenic transformation. In line with the findings illustrated above, knockdown of RHA expression critically reduced Ewing sarcoma cell viability, while no decrease in cell viability was detected in pancreatic (PANC1) and cervical (HeLa) tumor cell lines that do not express the EWS-FLI1 oncogene [91, 19]. Recently, it has been shown that RHA helicase activity is also affected by EWS-FLI1 binding, suggesting that a complex interplay between these proteins contributes to the pathogenesis of Ewing sarcomas [92].

### Development of synthetic peptides to interfere with RHA activity

As mentioned above, RHA is a critical gene for tumorigenesis. In the case of Ewing sarcoma, its oncogenic activity relies on its interaction with EWS-FLI1, which directly binds RHA in the distal portion of the helicase domain [86]. Since formation of the EWS-FLI1-RHA complex enhances EWS-FLI1 oncogenic activity and tumor maintenance [86], the possibility to modulate, or even inhibit, this interaction could have enormous therapeutic value in Ewing sarcoma. Recently, it has been shown that small molecule inhibitors are able to block RHA/EWS-FLI1 interaction (Figure 3). Taking advantage of the surface plasmon resonance (SPR) technique in a peptide screening, 3000 small molecules were tested for the binding to EWS-FLI1 [91]. The screen selected NSC635437 as lead compound for the binding to EWS-FLI1 and for its ability to reduce the binding between GST-RHA_(647-1075)_ and EWS-FLI1 [91]. Once this compound was identified, several analogs were designed with the aim to minimize its side effects and to improve its anti-oncogenic activity. One of them, YK-4-279, was developed by substituting the chlorine atom with a methoxyl group in position “para” of the aromatic ring of NSC635437. The resulting molecule was able to inhibit the interaction between RHA and EWS-FLI1 *in vitro* with a *K_d_* of 9.48 μM [91]. Remarkably, the molecule was also able to inhibit cell growth and to induce apoptosis in Ewing sarcoma cells, both *in vitro* and *in vivo* [91]. YK-4-279 mimics the structure of the E9R peptide, which corresponds to amino acids 823-832 located in the HA2 proximal region of RHA [91].

YK-4-279 structure contains a chiral center. To evaluate the specific effects of the two enantiomers, they were separated and tested individually or in racemic form, demonstrating that both YK-4-279 and (S)-YK-4-279 were able to prevent EWS-FLI1 mediated transcriptional activity, while the (R) enantiomer did not show any significant effect [93]. *In vivo* studies confirmed the *in vitro* data, demonstrating that the (S)-YK-4-279 form was the active enantiomer [94].

Recently, it has been demonstrated that EWS-FLI1 inhibits the helicase activity of RHA *in vitro* while YK-4-279 reverted EWS-FLI1 inhibitory effect thus restoring RHA helicase activity [92]. YK-4-279 displays its effect also toward other helicases. For example, the activity of p68 (DDX5), a RNA helicase that directly binds to EWS-FLI1, is affected by YK-4-279 treatment [90]. Moreover, when EWS-FLI1/RHA or EWS-FLI1/p68 complexes are impaired by YK-4-279 treatments, EWS-FLI1 mediated alternative splicing events are affected, although binding of EWS-FLI1 to the splicing regulators PRPF6 and hnRNP K is not influenced [90].

Given the expression of the *ets* genes *ERG* and *ETV1* in prostate cancers, YK-4-279 was also tested in prostate cancer cell lines, demonstrating its ability to reduce cell motility and tumor invasion [95].

### Genotoxic stress-induced alternative splicing to target RHA

High-throughput transcriptome analyses of Ewing sarcoma cells irradiated with UV light revealed a subset of genes and alternative splicing events that are regulated by this genotoxic stress [19]. Among them, the gene encoding RHA was affected both at gene expression and at the processing levels. In particular, it was identified a cassette exon event in the *RHA* transcript that is specifically induced by UV irradiation. This novel RHA splice isoform contained a premature stop codon (PTC) that targeted the transcript to nonsense-mediated RNA decay (NMD). As a consequence, in response to UV light treatment, the RHA protein was diminished in Ewing sarcoma cells [19]. The mechanism underlining this regulation involves the slowing down of the elongation rate of RNAPII to promote the inclusion of the alternative exon 6A in *RHA* pre-mRNA. This observation is in line with other studies showing that exons sensitive to RNAPII modulation often introduce PTCs that elicit NMD of the spliced mRNAs [96]. Thus, DNA damage represses RHA expression by alternative splicing through the inclusion of a novel PTC-containing exon and its consequent targeting to NMD [19]. Changes in expression of RHA modify the sensitivity of Ewing sarcoma cells to UV irradiation [19] (Figure 3). Notably, the chemotherapeutic agent etoposide efficiently suppressed Ewing sarcoma cell growth and was able to affect RNAPII phosphorylation and exon 6A splicing similarly to UV irradiation [19]. Thus, modulation of *RHA* splicing could be exploited as a new potential tool to enhance Ewing sarcoma cell sensitivity to genotoxic stresses.

## CONCLUDING REMARKS

Mounting evidence suggests that RHA is involved in several pathway strictly linked to cancer transformation and genomic instability. Interestingly, both the hinge role and the helicase property of RHA appear to be crucial in these processes. Indeed, CBP transcriptional activity is reduced both by deletion of the MTAD domain from RHA and by mutations that alter its ATPase activity (without modifying RNAPII binding) while the double mutant produces more severe effects [36]. This suggests that RHA regulates CBP pathway by both RNAPII recruitment and by its helicase activity. The importance of RHA helicase activity has been established also in Ewing sarcoma. It has been demonstrated that the oncoprotein EWS-FLI1 reduces RHA helicase activity causing changes in the transcription process and this may contribute to oncogenic mechanism [92]. Moreover, in Ewing sarcoma cells high levels of RHA are also required to withstand genotoxic insults. Upon UV light irradiation or treatment with etoposide, alternative splicing of *RHA* is modulated to induce a novel RNA isoform targeted to NMD, thus reducing RHA availability. This, in turn, affects EWS-FLI1 oncogenic activity, decreasing EWS-FLI1 target genes and determining an increase in cell apoptosis and a decrease in clonogenicity [19]. Interestingly, overexpression of RHA increases Ewing sarcoma resistance to genotoxic stress, but not non-sarcoma cancer cells [89, 19], indicating that the underlying mechanism involves RHA/EWS-FLI1 interaction and confirming that the activity of RHA is dependent on its molecular partners [91, 19].

The alterations of RHA (mutations or overexpression) observed in various tumors [16, 14, 39, 15] highlighted the involvement of this protein in cancer transformation and in sustaining genomic stability. Both functions of RHA are related to its interacting partners and to its intrinsic helicase activity. Given these premises, it is likely that understanding the mechanisms underlying RHA regulation would make it a good candidate for tumor therapy, especially in Ewing sarcoma where RHA roles has been extensively investigated [86, 91, 92, 19].

Ewing sarcomas often respond well to initial chemotherapy. Nevertheless, 40% of patients develop recurrent disease and die from Ewing sarcoma. Furthermore, 75-80% of patients who present at diagnosis with metastatic Ewing sarcoma will die within 5 years despite high-dose chemotherapy. Thus, alternative therapeutic approaches are urgently needed. Given the key role played by EWS-FLI1 in the disease, targeting its function is a promising approach. In this regard, the recent development of a small-molecule targeting EWS-FLI1-RHA interaction represents a good strategy to inhibit EWS-FLI1-mediated gene expression [90, 91] (Figure 3). Moreover, modulation of *RHA* expression levels by inducing a genotoxic stress-regulated NMD-targeted isoform could be a valuable additional tool to lower the expression levels of RHA, thus rendering Ewing sarcoma cells more sensitive to chemotherapeutic treatment [19]. In this scenario, the development of antisense oligonucleotides (ASOs) recruiting the spliceosomal complex to the alternative exon 6A in *RHA* could be instrumental to drive RHA downregulation in live cells and might provide a valuable additional therapy for the treatment of Ewing sarcoma. To this regard, preclinical evidence documents the efficacy of ASOs targeting the antiapoptotic splice variants of *BCL2L1* [98, 99] and *MDM4* [100] in cancer cells. Thus, a similar approach could offer new valuable perspective also in Ewing sarcoma.
